# The Triad of Risk: Linking MASLD, Cardiovascular Disease and Type 2 Diabetes; From Pathophysiology to Treatment

**DOI:** 10.3390/jcm14020428

**Published:** 2025-01-10

**Authors:** Eleni Michalopoulou, John Thymis, Stamatios Lampsas, George Pavlidis, Konstantinos Katogiannis, Dimitrios Vlachomitros, Eleni Katsanaki, Gavriella Kostelli, Sotirios Pililis, Loukia Pliouta, Aikaterini Kountouri, Ioannis S. Papanikolaou, Vaia Lambadiari, Ignatios Ikonomidis

**Affiliations:** 1 2nd Cardiology Department, Attikon University Hospital, National and Kapodistrian University of Athens, Rimini 1, Chaidari, 12462 Athens, Greece; elenimixa91@gmail.com (E.M.); johnythg@gmail.com (J.T.); geo_pavlidis@yahoo.gr (G.P.); kenndj89@gmail.com (K.K.); vlachomitrosdimitrios@gmail.com (D.V.); helenkatsanaki@gmail.com (E.K.); kosteligravriela@gmail.com (G.K.); 2 Diabetes Center, 2nd Department of Internal Medicine, Attikon University Hospital, Medical School, National and Kapodistrian University of Athens, Rimini 1, Chaidari, 12462 Athens, Greece; lampsas.stam@gmail.com (S.L.); sotiris181@yahoo.gr (S.P.); plioutaloukia@gmail.com (L.P.); katerinak90@hotmail.com (A.K.); vlambadiari@gmail.com (V.L.); 3 Hepatogastroenterology Unit, Second Department of Internal Medicine-Propaedeutic, Attikon University Hospital, Rimini 1, Chaidari, 12462 Athens, Greece; ispapn@hotmail.com

**Keywords:** metabolic dysfunction-associated fatty liver disease, diabetes mellitus, cardiovascular disease, pathophysiology, treatment, antidiabetic drugs

## Abstract

Metabolic dysfunction-associated steatotic liver disease (MASLD) is an emerging global health concern, and it is not only the keystone precursor of eventual liver-related morbidity, but it also places patients at considerably higher cardiovascular risk, which is still a leading cause of death in these patients. The most important common underlying pathophysiological mechanisms in these diseases are primarily related to insulin resistance, chronic inflammation and oxidative stress. The presence of MASLD with cardiovascular disease (CVD) and type 2 diabetes mellitus (T2DM) elevates the risk for poor outcomes, thus this review highlights a method to the therapeutic approaches. Given the intertwined nature of MASLD, T2DM, and CVD, there is an urgent need for therapeutic strategies that address all three conditions. Although lifestyle changes are important as treatment, medication plays a crucial role in managing hyperglycemia, enhancing liver function and lowering cardiovascular risk. The onset and progression of MASLD should be addressed through a multifaceted therapeutic approach, targeting inflammatory, immune, metabolic, oxidative stress, hormonal and gutaxis pathways, alongside the treatment strategies for T2DM. In this review, we discuss the effects of antidiabetic drugs with an impact on both liver outcomes and cardiovascular risk in patients affected by MASLD, T2DM and CDV.

## 1. Introduction

**Figure 1:**
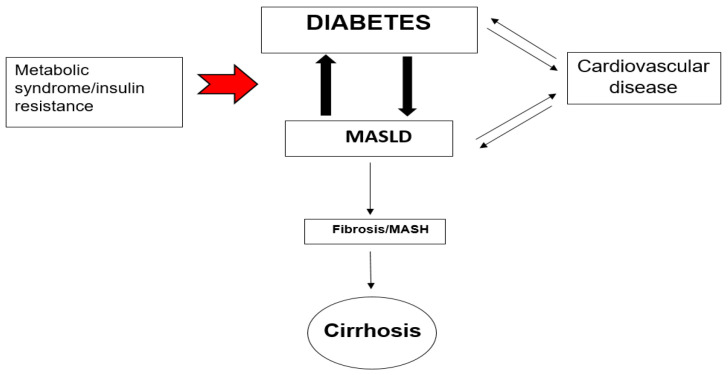
The triad of risk: Metabolic dysfunction-associated steatotic liver disease fatty liver disease (MASLD), diabetes mellitus, and cardiovascular disease (common CVD like atherosclerosis, myocardial infarction, heart failure, arrhythmias which are associated with diabetes mellitus and MASLD), metabolic-associated steatotic liver disease steatohepatitis (MASH). The red arrow shows that the metabolic syndrome and insulin resistance are main causes for the strong interaction and association of MASLD and Diabetes (big black arrows). The thin single black arrows show the path-effect while the double ones black arrows the interaction between the conditions.

Metabolic dysfunction-associated steatotic liver disease (MASLD) is characterized by hepatic fat content, primarily triglycerides, exceeding 5% of liver weight, without other identifiable causes of liver steatosis or chronic liver disease. MASLD ranges from simple or isolated steatosis to metabolic dysfunction-associated steatohepatitis (MASH) [1,2], which is characterized by the histological presence of steatosis, inflammation and hepatocyte ballooning, with or without fibrosis [3], and can finally lead to cirrhosis and to hepatocellular carcinoma (HCC) [4,5]. In the United States, MASH is recognized as a leading cause of cirrhosis and cirrhosis due to MASH is now the second most common reason for liver transplantation [6]. MASLD is the most common liver disorder globally and while it was formally described over 40 years ago, it is only in recent years that it has become recognized as an urgent unmet medical need [7], affecting 25% of the adult population and 50–70% individuals with type 2 diabetes (T2DM). Diabetes and obesity are always linked to ectopic fat accumulation in the liver, a condition referred to as metabolic dysfunction-associated steatotic liver disease (MASLD), previously known as NAFLD. A more serious issue is the progression to steatohepatitis, seen in approximately one-third of individuals with excess weight, termed metabolic dysfunction-associated steatohepatitis (MASH), formerly known as multifaceted abstruse nature steatohepatitis. Those with MASH face an approximately 10% risk of developing cirrhosis and advanced liver disease [8,9,10]. This association is driven by insulin resistance (IR), a central feature of both MASLD and T2DM. Patients with MASLD and T2DM have a 1.5- to 2-fold increased risk of myocardial infarction, stroke, as well as other cardiovascular events than those without MAFLD. MASLD has since gained recognition as an independent risk factor for the development of cardiovascular disease (CVD), which is now widely considered to be the main cause of morbidity and mortality in MASLD [11,12]. Heart disease and cardiovascular complications including coronary heart disease, heart failure, aortic valve sclerosis, atrial fibrillation, rather the progress of liver disease, are the leading cause of death in MASLD patients. A population-based cohort study in Minnesota, United States, with an average follow-up of 7.6 years, found that ischemic heart disease (IHD) accounted for 25% of deaths among MASLD patients, nearly double the mortality rate from liver disease [13]. Large cohort studies have shown that the existence of MASLD in patients with T2DM increases the risk of cardiovascular events independently of traditional risk factors such as hypertension, smoking, and hyperlipidemia. The mechanisms driving this increased risk contains a pro-inflammatory, pro-thrombotic state, endothelial dysfunction, and accelerated atherosclerosis [14,15] and the stage of fibrosis is linked with the incidence of CVD and cardiovascular (CV) mortality. Among MASLD patients, 20% already have cardiovascular complications and 60% higher risk of developing cardiovascular events [11]. The EASL-EASD-EASO Clinical Practice Guidelines recommend that patients with MASLD be assessed for cardiovascular risk factors, given that cardiovascular disease is the leading cause of mortality in this population. This triad of MASLD, T2DM and CVD is a challenge from a clinical standpoint with such patients doing markedly worse. Given the tight linkage between these conditions, mechanisms that improve liver-related and cardiovascular outcomes in addition to improving glycemic control are warranted (Figure 1).

## 2. Pathophysiological Link Between MASLD, T2DM and CVD

### 2.1. Genetic Factors

Genetic predisposition is apparent in the pathogenesis of ΜASLD, where several gene polymorphisms (e.g., PNPLA3 and TM6SF2) are associated with greater liver fat accumulation and severity of fibrosis. These genetic factors make some individuals particularly vulnerable to a severe form of liver disease when they become insulin resistant. The PNPLA3 gene encodes adiponutrin, a triglyceride (TG) lipase that regulates TG and retinoid metabolism. The PNPLA3 I148M variant resists proteasomal degradation and accumulates on lipid droplets, disrupting lipolysis and altering phospholipid remodeling. This variant (PNPLA3 SNP rs738409) is strongly linked to hepatic steatosis, steatohepatitis, fibrosis, and hepatocellular carcinoma (HCC). TM6SF2, another gene, plays a role in very low-density lipoprotein (VLDL) secretion from liver cells, resulting in higher liver TG content and lower circulating lipoproteins. Both PNPLA3 and TM6SF2 variants are associated with increased hepatic steatosis, more severe MASH, and advanced fibrosis/cirrhosis [16,17]. Interestingly, TM6SF2’s role in promoting VLDL secretion also heightens the risk of dyslipidemia and cardiovascular disease (CVD) [18]. The eight hub genes associated with type 2 diabetes and NASH are IL-6, FOS, GADD45B, NR4A1, FOSB, MYC, NR4A2, and GADD45G. These genes offer new strategies and potential targets for future treatments of NASH induced by type 2 diabetes [19].

### 2.2. Insulin Resistance, Lipotoxicity

Genetic and epigenetic factors play a role in the pathogenesis of MASLD, supporting the “multiple parallel-hit” model, in which various “hits” interact dynamically to drive the onset and progression of the disease [20]. MASLD seems to be extremely common in patients with T2DM. This issue is unsurprising, as insulin resistance, a hallmark of T2DM, is central to MASLD pathogenesis [20]. Insulin resistance is frequently identified as a key factor in this context and is widely regarded as a fundamental cause of the metabolic syndrome, as well as its related conditions, including metabolic dysfunction-associated fatty liver disease, polycystic ovary syndrome (PCOS), T2DM, obesity and atherosclerotic cardiovascular disease (ASCVD) [21]. In ΜASLD patients, increased visceral adipose tissue and uninhibited hormone-sensitive lipase activity enhance triglyceride hydrolysis, raising free fatty acid (FFA) levels, especially in the portal vein, which in turn increases FFA uptake by the liver [22]. Reduced glucose uptake by skeletal muscle further promotes lipid accumulation in hepatocytes [23]. However, the liver retains partial insulin sensitivity, as hepatic lipogenesis remains responsive to insulin, even in severe insulin resistance, which contributes to increased FFA influx into the liver [24,25]. Additionally, hyperinsulinemia lowers apo-lipoprotein-B production, reducing VLDL-associated lipid export from the liver, which results in hepatic triglyceride accumulation and limits triglyceride secretion as VLDL (very low-density lipoprotein) [24]. This process, known as hepatic de novo lipogenesis, generates toxic metabolites like glycerol and ceramides, perpetuating insulin resistance and creating a cycle that exacerbates hepatic steatosis [26]. Accumulation of hepatic fat can lead to lipotoxicity, oxidative stress, and inflammation and drive progression from simple steatosis through MASH ultimately to fibrosis over time [27].

### 2.3. Inflammation, Cytokines, Oxidative Stress

Oxidative stress is closely linked to metabolic dysfunction-associated steatotic liver disease (ΜASLD). Oxidative stress represents an imbalance between reactive oxygen species (ROS) and the body’s ability to neutralize them with antioxidants [28]. Under normal conditions, ROS levels are regulated to maintain homeostasis, which is essential for physiological redox reacting signaling. At normal levels, ROS function plays roles in cellular processes, including metabolism, survival, immune response, proliferation, and differentiation, by influencing transcription factors and epigenetic mechanisms [29]. However, when oxidative stress occurs, excessive ROS can lead to harmful redox reacting signaling, resulting in cellular damage associated with multiple diseases, especially when the production of ROS shifts to more toxic forms [30,31]. Even slight increases in ROS can cause cell toxicity and oxidative stress. Excess ROS can result in lipid peroxidation, enhanced mitochondrial and peroxisome fatty acid oxidation, and the release of cytokines [32]. Additionally, elevated ROS levels and oxidative stress are thought to be key factors in the development of insulin resistance [33,34]. The liver is a vital metabolic organ that may contribute to systemic inflammation by releasing inflammatory markers, chemokines, and cytokines into circulation. These inflammatory agents can lead to cardiovascular (CV) complications through mechanisms such as endothelial dysfunction, plaque formation, changes in vascular tone and altered coagulation. Several inflammatory markers, including tumor necrosis factor (TNF)-α, interleukin (IL)-6, CCL3, soluble intercellular adhesion molecule-1 (sICAM-1), and C-reactive protein (CRP), as well as elevated hepatic expression of IL-6, TNF, CXCL10, and IL1RN, have been linked to fatty liver disease (FLD) and increased CV risk [35,36]. Increasing levels of inflammation and insulin resistance (IR) are linked to poorer cardiometabolic outcomes as the severity of metabolic dysfunction-associated steatotic liver disease (ΜASLD) progresses. While oxidation is a vital mechanism for the body to combat infections, oxidative stress occurs due to changes in free radical and antioxidant activities. This imbalance can trigger various diseases due to the abnormal release of cytokines such as TNF-α and IL-6 [37,38]. Research indicates that oxidative stress may play a role in the development of cardiovascular disease (CVD) in individuals with fatty liver disease, also contributing to the worsening of liver disease from simple fat accumulation to steatohepatitis [39].

### 2.4. ΜAFLD and CVD

**Figure 2:**
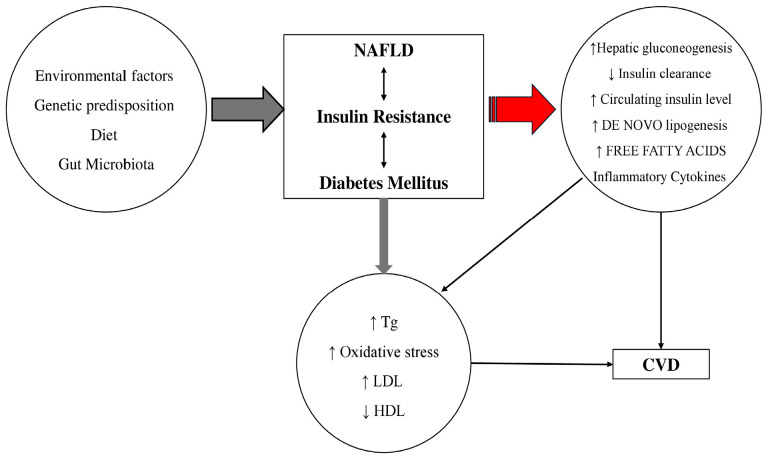
The pathophysiological link between metabolic dysfunction-associated steatotic liver disease (MASLD) (previously known as NAFLD: Nonalcoholic fatty liver disease), diabetes mellitus and cardiovascular disease (CVD). Tg: triglycerides; LDL: low-density lipoprotein; HDL: high-density lipoproteins. Insulin resistance is associated with an increase infree fatty acids that contributes to increased TG production that stimulates secretion of LDL in hepatocytes. Fat accumulation in the liver is associated with oxidative stress and lipid peroxidation. Additionally, an increased secretion of inflammatory markers and plasma glucose and a decrease in HDL concentration have been observed in MASLD patients. The result of this physiological dysfunction is increased risk for the development of diabetes and atherosclerosis and increased risk for coronary artery disease. The single arrows regardless of color and thickness mean “leads to”, the double thin black arrows indicate the interaction.

The causal link between metabolic dysfunction-associated steatotic liver disease (ΜASLD) and cardiovascular disease (CVD) is well recognized, supported by various pathophysiological mechanisms that connect the two conditions. These mechanisms include endothelial dysfunction, altered lipid metabolism, systemic inflammation, plaque formation and instability, oxidative stress, and insulin resistance [40]. Ultimately, the disruption of the structure, function, and electrical activity of the cardiovascular system (CVS) increases the risk of developing CVD in patients with ΜASLD, leading to complications such as hypertension, atherosclerosis, arrhythmias, myocardial dysfunction, cardiac valve deformities, and venous thrombosis. ΜASLD is associated, due to insulin resistance, with dyslipidemia, characterized by increased levels of small dense LDL, elevated triglycerides, and reduced HDL cholesterol [41,42]. These lipid abnormalities, coupled with systemic inflammation, contribute to endothelial dysfunction and the accelerated development of atherosclerosis [43]. Atherosclerosis that occurs alongside metabolic dysfunction-associated fatty liver disease or metabolic dysfunction-associated steatohepatitis presents a greater metabolic burden than atherosclerosis on its own. In this regard, several studies have shown a significant link between ΜASH and: (1) carotid atherosclerosis and (2) subclinical signs of atherosclerosis in patients with or without type 2 diabetes (T2DM) [44]. These signs include increased intima-media thickness, endothelial dysfunction, arterial stiffness, impaired left ventricular function, reduced flow-mediated vasodilation, and coronary calcification [45]. In patients with both ΜASLD and T2D, the presence of insulin resistance amplifies these cardiovascular risk factors, leading to a higher incidence of cardiovascular events [43] (Figure 2). Identifying individuals with ΜASLD could also help pinpoint a subgroup of diabetic patients who would benefit from more intensive treatment to reduce their risk of future cardiovascular events [46].

### 2.5. The Gut Microbiota

The gut microbiota functions as an “invisible organ” within the body and plays a crucial role in metabolism and immune regulation. The gut microbiota provides benefits, including aiding in nutrient and energy absorption, as well as producing molecules like short-chain fatty acids (SCFAs), secondary bile acids (BAs), and choline derivatives, which can influence host metabolism through various molecular pathways. The composition of the gut microbiota can change due to factors such as high-fat diets (HFD), antibiotic use, or exposure to toxins. The gut microbiota has become recognized as an important factor in the development and progression of metabolic dysfunction-associated steatotic liver disease (ΜASLD). The gut and liver communicate with each other through the portal vein, biliary tract, and systemic circulation. Intestinal products, such as host and/or microbial metabolites and microbial-associated molecular patterns (MAMPs), are transported to the liver through the portal vein and influence liver function [47]. The gut and liver are interconnected beyond just the portal vein; growing evidence suggests that metabolites produced by gut microbiota can trigger a significant inflammatory response in the liver, primarily through the activation of Kupffer cells [47]. In fact, patients with metabolic dysfunction-associated steatotic liver disease (ΜASLD) often exhibit elevated levels of lipopolysaccharides (LPS) [48]. ΜASLD has been linked to increased gut permeability, small intestinal bacterial overgrowth, and endotoxemia, with lipopolysaccharide (LPS) being a key contributor. LPS, a component of Gram-negative bacteria, is found in elevated levels in the intestines of patients with ΜASH. It impacts insulin signaling via the Toll-like receptor 4 and monocyte differentiation antigen CD14 pathway, resulting in insulin resistance. Additionally, LPS can activate inflammasomes, which contributes to ongoing hepatic inflammation [49]. Recent studies have also demonstrated a significant link between gut microbiota and metabolic dysfunction-associated steatotic liver disease (ΜASLD) in both mice and humans. ΜASLD is associated with changes in the gut microbiota profile, and the development of chronic liver disease is exacerbated by the overgrowth of certain bacteria in the small intestine [50]. Additionally, changes in the composition of gut microbiome have been linked to pathologies such as atherosclerosis, hypertension, heart failure, chronic kidney disease, obesity and type 2 diabetes mellitus. Leaky gut, changes in the microbiome, and reduced ability to clear toxins have been linked to the progression of ΜASLD [51].

## 3. Monitoring Patients with ΜASLD

**Figure 3:**
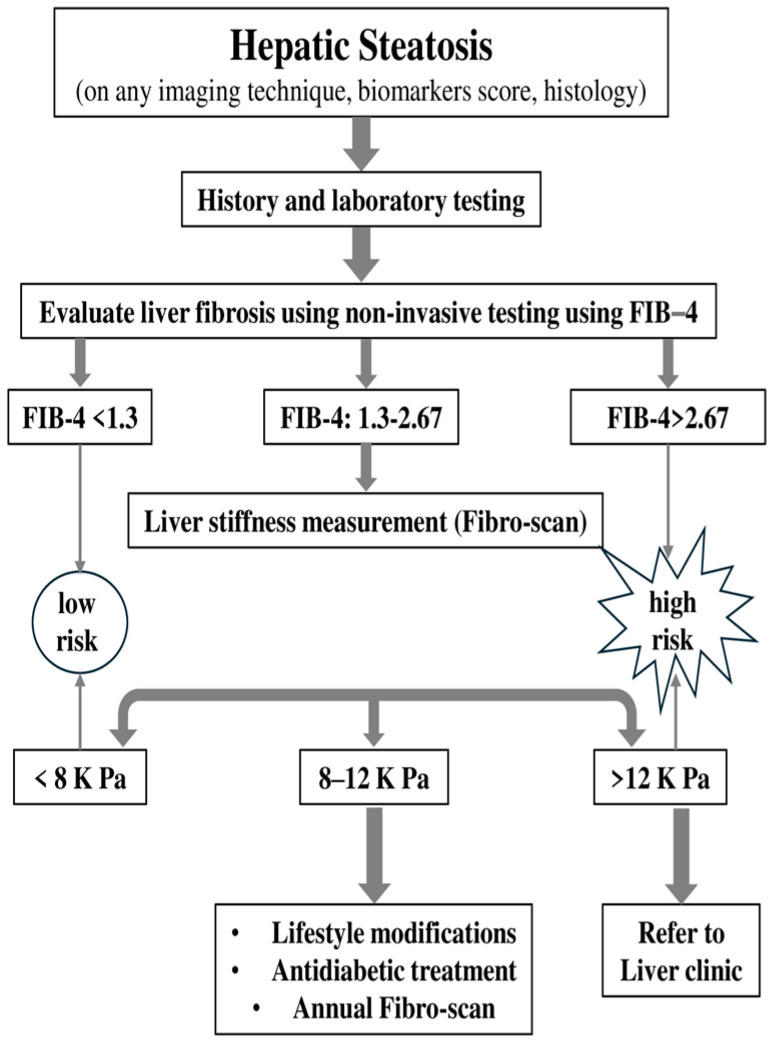
Algorithm of monitoring based on FIB-4 (FIB-4: fibrosis-4).

Diagnosing ΜASLD/ΜASH continues to be a challenge today. However, the future looks promising as screening that utilizes a combination of validated genetic, imaging, and plasma biomarkers will help improve management. It is an exciting era where increased awareness of the effects of MASH will lead to earlier diagnoses and treatments that could potentially change the disease’s progression and enhance the quality of life for millions of patients [52]. To diagnose MASLD, it is essential to rule out alcohol-related liver disease, which requires confirming an alcohol intake of less than 20–40 g per day [53]. Routine screening for MASLD in the general population is not advised but due to the well-established link between type 2 diabetes (T2DM) and metabolic dysfunction-associated steatotic liver disease (MASLD), the American Diabetes Association (ADA) and the American Association for the Study of Liver Diseases (AASLD) advise screening all T2DM patients for MASLD [50] and its more advanced form, metabolic dysfunction-associated steatohepatitis (MASH) [54]. The Asian Pacific Association for the Study of the Liver (APASL) guidelines, also, for metabolic-associated steatotic liver disease (MASLD) advise that patients be monitored at regular intervals, with the frequency determined by the degree of hepatic fibrosis identified at baseline [54]. Among those diagnosed with MASLD, only patients at higher risk for developing complications require further evaluation and regular monitoring, as only a small percentage will face severe clinical events. The European Association for the Study of Liver Disease (EASL) recommends noninvasive initial assessments for individuals with metabolic syndrome, obesity, and type 2 diabetes mellitus (T2DM). Additionally, the American Diabetes Association (ADA) suggests annual liver transaminase assessments for those with T2DM. Liver biopsy is considered the gold standard for diagnosing MASH, but it has its drawbacks. As a result, there is an increasing use of noninvasive biomarkers [55]. However, relying solely on transaminases for screening is inadequate, as it may overlook a significant number of patients with advanced liver fibrosis (F2 or higher) having normal transaminase levels [21]. Patients at risk for advanced fibrosis include those with two or more metabolic risk factors as well as those with type 2 diabetes, identified hepatic steatosis, or elevated liver enzymes. For these individuals, a comprehensive history and laboratory tests should be conducted if they have not been already. These data can then be used to calculate the fibrosis-4 index (FIB-4), which estimates fibrosis risk based on age, AST, ALT, and platelet count. The development of less invasive and more consistent standards could speed up the approval of effective therapeutics [56]. The FIB-4 score helps classify patients into three risk categories: low risk (<1.3), indeterminate risk (1.3–2.67), and high risk (>2.67) for advanced fibrosis. For those at low risk, the American Gastroenterological Association (AGA) suggests retesting, including recalculating FIB-4, every 2–3 years, unless clinical changes occur. Patients at high risk should be referred directly to a liver expert. For those in the indeterminate category, further assessment with liver stiffness measurement (LSM) is recommended to refine risk classification. LSM results categorize patients into low (<8 kPa), indeterminate (8–12 kPa), and high (>12 kPa) risk groups (Figure 3).

Patients classified as low or high risk based on LSM should follow the same guidelines as those in similar FIB-4 categories, while those at indeterminate risk on LSM should either be referred to a liver expert or undergo re-evaluation in 2–3 years [57]. Transient elastography (TE) boasts high applicability in patients without severe obesity, is straightforward to perform, and offers immediate results. Increased liver stiffness values, are generally associated with liver fibrosis, though they may also rise due to other factors, such as inflammation, food intake, exercise. Magnetic resonance elastography (MRE), on the other hand, can typically be performed using standard MRI equipment. Unlike TE, MRE evaluates the entire liver, leading to more comprehensive and robust results. MRE also demonstrates better applicability than TE in challenging conditions, such as in patients with ascites or obesity. This broader applicability makes MRE an advantageous alternative for liver fibrosis assessment in certain populations [58]. Portal hypertension is a common complication in all chronic liver diseases as they advance to cirrhosis. Observational clinical studies indicate that while most patients with MASLD who develop gastroesophageal varices typically have advanced fibrosis (F3 or F4), a smaller subset (approximately 20%) may experience portal hypertension even at earlier stages of fibrosis, such as F1 or F2 [59]. The Baveno VI Consensus Workshop concluded that patients with liver stiffness below 20 kPa and a platelet count over 150,000 have a very low risk of having varices that require treatment. As a result, these patients may safely avoid screening endoscopy and instead be monitored with annual follow-up using TE and platelet count measurements [59,60,61].

## 4. Current Therapeutic Approaches for Managing NAFLD-MASLD, T2DM, and CVD Risk Factors

### 4.1. Lifestyle Modifications

Even though various treatment strategies are currently available or being studied for MASLD and T2D, lifestyle modifications still represent the mainstay of management, with weight loss resulting in the effective resolution of liver histology as well as substantial cardiovascular risk reduction. The AASLD Guidelines suggest that weight reduction of 7–10% is important for substantial improvement in MASH, decrease liver fat and has been shown to significantly improve liver histology, including hepatic steatosis, inflammation, and fibrosis. Long-term weight loss also has the potential to reverse steatosis and improve insulin sensitivity, reduce inflammation as well [62]. Obesity exacerbates liver fat accumulation indirectly by reducing the production of adiponectin in adipose tissue, which leads to decreased fatty acid oxidation in the liver [63]. The Mediterranean diet, rich in fruits and vegetables, whole grains and healthy fats has shown important benefits on the amount of hepatic fat content; it is able to improve metabolic parameters both in patients affected by MASLD or T2DM. A randomized controlled trial showed that the Mediterranean diet, even without significant weight loss, led to a reduction in liver fat and improvements in glycemic control in MASLD patients: Not have only low-carbohydrate and ketogenic diets have also been effective in reducing liver fat and improving metabolic parameters but also high-protein intake has beneficial effects on liver fat content in patients with MASLD [62]. These diets deliver a greater improvement in fat oxidation and insulin sensitivity that might explain the significant decrease in the hepatic steatosis and central adiposity which are crucial aspects for MASLD/T2DM management [63]. The Nutrient Hazard Analysis and Critical Control Point (NACCP) process offers a systematic approach to monitoring the nutritional quality of foods within a chosen dietary pattern, ensuring that they contain the highest levels of antioxidants [64]. Intermittent fasting may have beneficial effects on liver fat content, insulin sensitivity, and cardiovascular risk factors. Intermittent fasting regimens, such as time-restricted feeding and alternate-day fasting, have been shown to reduce hepatic triglyceride content and improve lipid profiles, making them a potential adjunct to conventional dietary interventions. Physical activity, particularly aerobic exercise, improves insulin sensitivity and reduces liver fat in patients with MASLD. Physical activity, whether aerobic or resistance based, should be strongly encouraged for managing fatty liver in patients with type 2 diabetes, as the benefits extend beyond just weight loss [65]. Although the effects of exercise on weight reduction and hepatic fat loss are not entirely clear, it has been demonstrated that exercise can improve liver enzyme levels and insulin resistance in all diabetic patients with MASLD and MASH [66]. Additionally, physical activity is beneficial in reducing cardiovascular risk, improving endothelial function [67]. In a sub-analysis of the LOOK-AHEAD study conducted after 12 months, participants who were assigned to an intensive lifestyle intervention experienced a significantly greater weight loss compared to those who received only therapeutic support and education. Additionally, they exhibited a more substantial reduction in hepatic steatosis [68]. In contrary for patients with type 2 diabetes and MASLD, pharmacological treatments are more effective than lifestyle changes for managing glucose levels. Furthermore, combining lifestyle modifications with antidiabetic medications is likely to have a synergistic effect, reducing cardiovascular risk factors and decreasing hepatic fat accumulation. This combination can help delay the progression of inflammation and fibrosis in these patients [69].

### 4.2. Vitamin E

Before 2000, the acknowledgment of vitamin E’s antioxidant properties, along with emerging evidence linking lipoprotein oxidation to atherogenesis, created excitement about using vitamin E as a natural approach to prevent atherosclerosis. This enthusiasm prompted extensive cardiovascular outcome studies, which ultimately showed that taking vitamin E in doses of 300–400 IU per day did not have significant beneficial or harmful effects on cardiovascular events [70]. Guidelines recommend the use of vitamin E for non-diabetic individuals who have been diagnosed with MASH confirmed by biopsy [71]. PIVENS and several clinical studies have investigated the use of vitamin E in both adult and pediatric patients with metabolic dysfunction-associated steatotic liver disease (MASLD), metabolic dysfunction-associated steatohepatitis (MASH), and/or type 2 diabetes (T2D) [72]. Many of these studies observed reductions in alanine aminotransferase (ALT) levels and/or improvements in hepatic steatosis or MASH, either alone or as part of combination therapies, findings that have been supported by a meta-analysis [73]. Recent findings indicate that the haptoglobin 2 allele is linked to a histological response to vitamin E treatment in patients with metabolic dysfunction-associated steatohepatitis (MASH). This suggests that genotyping for the haptoglobin allele could help identify individuals who are more likely to benefit from vitamin E therapy. Additionally, the haptoglobin 2 allele has been associated with a decrease in cardiovascular disease (CVD) risk among individuals with type 2 diabetes (T2D) who received vitamin E [74]. This association was demonstrated in several studies, including the Heart Outcome Prevention Evaluation (HOPE) study, the Women’s Health Study, and the Israel Cardiovascular Vitamin E (ICARE) study [75].

### 4.3. Lipid-Lowering Therapies

Lipid-lowering therapies are central in the management of patients with metabolic dysfunction-associated fatty liver disease (MASLD), type 2 diabetes mellitus (T2DM) and high cardiovascular risk. The risk of cardiovascular disease (CVD) associated with MASLD, is closely related to dyslipidemia and metabolic syndrome and that issue highlights the significance of lipid control. There has been a longstanding belief that polyunsaturated fatty acids (PUFAs) can reduce hepatic steatosis and suppress pro-inflammatory pathways in MASLD/MASH by activating peroxisome proliferator-activated receptor (PPAR) α receptors and enhancing the expression of several genes involved in fatty acid oxidation. This view was supported by small, uncontrolled studies showing reductions in plasma aminotransferases and liver steatosis. However, more recent randomized controlled trials (RCTs) have mostly produced negative findings, indicating that PUFAs may not have a major role in the management of MASH [76]. Statins are the first-choice drugs for dyslipidemia and one of the best tested agents in terms of cardiovascular benefits, at least in this population. Hydroxy-3-methyl-glutaryl-coenzyme A (HMG-CoA) is the rate-limiting enzyme involved in cholesterol synthesis in the liver. Elevated levels of HMG-CoA in patients with metabolic dysfunction-associated fatty liver disease (MASLD) are linked to common comorbidities, including obesity, hyperlipidemia, and increased cardiovascular mortality. Statins, which are inhibitors of HMG-CoA reductase, are used to lower low-density lipoprotein (LDL) cholesterol and triglycerides while raising high-density lipoprotein (HDL) levels. Additionally, their anti-inflammatory properties and ability to reduce oxidative stress suggest that statins may offer benefits for patients with MASLD [77,78]. While there always concerns regarding potential hepatotoxicity with initial experience, current guidelines and studies have shown statins to be safe in patients with MASLD without a significant rise of liver enzymes or deterioration of hepatic function. The various subclasses of statins exhibit different characteristics in terms of oral absorption, bioavailability, liver extraction, protein binding, and activity on HMG-CoA reductase. Atorvastatin, lovastatin, simvastatin, and fluvastatin are classified as lipophilic statins and are metabolized by the cytochrome P450 enzyme system. In contrast, pravastatin and pitavastatin are hydrophilic, resulting in minimal hepatic metabolism, while rosuvastatin displays intermediate properties [14]. Research has indicated that the hydrophobic statins, such as lovastatin and simvastatin, achieve higher concentrations in the liver compared to the hydrophilic statin pravastatin [14]. Data from the GREACE study indicate that, indeed it is possible statins may reduce hepatic inflammation and fibrosis progression in addition to demonstrating favorable effects on CVD risk profile without damaging liver function when used for treatment of MASLD [79,80]. The St. Francis Heart Study demonstrated a reduction in steatosis on radiological imaging with a daily dosage of 20 mg of atorvastatin combined with vitamins C and E [81]. Additionally, a study involving patients with type 2 diabetes mellitus (T2DM) and metabolic dysfunction-associated steatohepatitis (MASH) found that those using statins had lower levels of alanine aminotransferase (ALT) and aspartate aminotransferase (AST) compared to non-users after 36 months [82,83]. In a multicenter, prospective study conducted with 107 individuals across Europe, statins demonstrated a protective effect on the NAFLD Activity Score (NAS) (*p* < 0.001) and on fibrosis stages F2–F4 (*p* = 0.017). Furthermore, improvements in MASH and associated histological lesions were found to correlate strongly with the dosage of statins administered [84,85]. The guidelines from the European Association for the Study of the Liver (EASL), the European Association for the Study of Diabetes, and the European Association for the Study of Obesity do not recommend the use of statins specifically for the treatment of metabolic dysfunction-associated steatotic liver disease (MASLD) or metabolic dysfunction-associated steatohepatitis (MASH). However, they indicate that statins can be safely prescribed to patients with MASLD/MASH who also have dyslipidemia [14]. Generally, the current guidelines (including that of American Association for the Study of Liver Diseases and FDA) recommend statins as first-line agents either alone or in combination with other drugs based on consent over individualized lipid profiles and risk factor assessment [86]. Fibrates are effective in treating hypertriglyceridemia, which is common in MASLD and T2DM, as noted in the ACCORD-Lipid trial, which demonstrated that fibrates could reduce cardiovascular risk when combined with statins in diabetic patients with elevated triglycerides [87]. In mouse models of metabolic dysfunction-associated steatohepatitis (MASH), activation of peroxisome proliferator-activated receptor alpha (PPARα) has been shown to exert anti-inflammatory effects and may help address various stages of metabolic dysfunction-associated steatotic liver disease (MASLD) [87]. PPARα activation lowers plasma triglyceride levels and increases high-density lipoprotein cholesterol (HDL-C) through multiple mechanisms, including enhanced hepatic beta-oxidation, induction of lipoprotein lipase, modulation of hepatic lipogenesis, and stimulation of the synthesis of major HDL-C apolipoproteins, specifically apolipoproteins AI and AII [76]. PCSK9 inhibitors, such as alirocumab and evolocumab, provide additional LDL-C reduction in cases of statin intolerance or inadequate response, and have been shown to significantly reduce cardiovascular events in high-risk populations, as seen in the FOURIER and ODYSSEY trials [88,89] (Table 1).

### 4.4. Blood Pressure Control

Blood pressure (BP) control is integral in managing patients with MASLD, type 2 diabetes, and cardiovascular comorbidities. Hypertension is highly prevalent in this cohort and is a major driver of both cardiovascular disease and the progression of MASLD to more advanced stages, such as metabolic dysfunction-associated steatohepatitis (MASH), cirrhosis, and liver fibrosis. The pathophysiological overlap between MASLD, T2DM, and hypertension, primarily mediated through insulin resistance and chronic inflammation, necessitates stringent BP control to prevent end-organ damage [86]. Hypertension is primarily an asymptomatic condition that is often identified through screening programs or incidental blood pressure measurements. Consequently, individuals with metabolic dysfunction-associated steatotic liver disease (MASLD) should be screened for hypertension [90]. Studies such as the Framingham Heart Study have highlighted that individuals with metabolic syndrome, including MASLD, are at a higher risk of hypertension-related cardiovascular events [91]. Accordingly, the American Heart Association (AHA) and European Society of Cardiology (ESC) recommend a target BP of <130/80 mmHg for patients with T2DM and cardiovascular risk factors. Among anti-hypertensive therapies, angiotensin-converting enzyme (ACE) inhibitors and angiotensin receptor blockers (ARBs) are considered the cornerstone due to their nephron-protective properties, particularly important in diabetes, and their potential to reduce hepatic inflammation and fibrosis progression, as shown in various clinical studies [86,92]. Angiotensin receptor blockers (ARBs) are widely recognized for their effectiveness in managing hypertension, which is a significant aspect of metabolic syndrome. Experimental studies have demonstrated that angiotensin II enhances the survival of hepatic myo-fibroblasts through the activation of IkB kinase, leading to the phosphorylation of the NF-kB subunit RelA [93]. A small pilot study involving seven patients with metabolic dysfunction-associated steatohepatitis (MASH) who were treated with losartan for 48 weeks revealed improvements in both necro-inflammation and fibrosis [94]. The RENAAL study, for instance, demonstrated the long-term renal and cardiovascular benefits of ARB therapy in diabetic patients. In addition, ARBs have shown potential anti-fibrotic effects in MASLD by inhibiting pro-inflammatory pathways and reducing oxidative stress within the liver [95]. Other anti-hypertensives, such as calcium channel blockers and beta-blockers, may be added based on patient-specific needs, although ACE inhibitors and ARBs remain first-line therapy due to their dual renal and cardiovascular protective effects. Consistent evidence indicates that targeting the renin-angiotensin system (RAS) in metabolic dysfunction-associated steatotic liver disease (MASLD) can affect adipogenesis, as well as the production of adipokines and cytokines. It also interacts with insulin receptors and intracellular signaling pathways, potentially influencing pancreatic β-cell insulin secretion [95,96]. The local hepatic effects are primarily mediated by angiotensin II receptor 1 (AT1R), which is found in hepatocytes, bile duct cells, hepatic stellate cells, and vascular endothelial cells, where it facilitates the actions of angiotensin II in the liver. Conversely, angiotensin II receptor 2 (AT2R) is associated with antifibrogenic effects [96]. Therefore, inhibiting the RAS could enhance the activity of intracellular signaling pathways, regulate adipose tissue proliferation, modulate adipokine production, and promote a more stable release of cytokines and chemotactic factors [90].

### 4.5. Metformin: The First-Line Drug

Metformin is an effective pharmacologic treatment for T2DM, which acts on the dual mechanism of improving insulin sensitivity and reducing hepatic glucose production; it has been used as a first-line drug [97]. Metformin positively impacts glucose and lipid metabolism. On a molecular level, it works by inhibiting the mitochondrial respiratory chain, which leads to a temporary decrease in cellular energy. This reduction activates adenosine monophosphate-activated protein kinase (AMPK), an essential regulator of glucose and lipid metabolism [98]. AMPK activation subsequently suppresses gluconeogenesis and lipogenesis in the liver, enhances glucose uptake in muscle tissue, and boosts fatty acid oxidation in both the liver and adipose tissue. In adipose tissue, metformin also reduces lipolysis and influences the synthesis and/or release of adipokines [99]. Metformin has been the subject of multiple randomized controlled trials, which have indicated that metformin is appropriate for lowering blood glucose levels and body weight compared to placebo or other anti-hyperglycemic therapies as well reducing cardiovascular event risk among people with T2DM [100]. While it is not specifically be approved for the treatment of MASLD, various studies have examined its benefits in these patients, especially due to its ability to improve insulin sensitivity and reduce hepatic fat accumulation [68]. By improving insulin sensitivity, metformin may mitigate these processes. Several studies, such as the TONIC trial, have demonstrated that metformin reduces hepatic steatosis and liver enzyme levels, although its impact on more advanced stages of liver fibrosis is not clear [101]. Additionally, it is mentioned that metformin improves liver function tests and reduces liver fat content in patients with MASLD and T2DM, though it is less effective in preventing fibrosis progression [102]. Moreover, metformin is recommended by guidelines as one of the available first-line therapies for managing hyperglycemia in T2DM patients, many of whom also have MASLD [100]. The ADA emphasizes metformin’s cardiovascular benefits, which are supported by data from studies such as the UK Prospective Diabetes Study (UKPDS) and subsequent trials like HOME. These studies showed that metformin significantly reduces the incidence of cardiovascular events and mortality in overweight patients with T2DM [103]. Metformin’s cardiovascular protective effects are attributed to its ability to reduce systemic inflammation, improve endothelial function, and decrease atherogenesis factors that are crucial in the context of both MASLD and T2DM, where patients are at heightened risk of cardiovascular disease (CVD) [100]. In addition to its direct effects on glycemic control and cardiovascular protection, metformin has been studied for its role in reducing the progression of MASLD. However, current evidence is mixed regarding its efficacy in reversing liver fibrosis or preventing cirrhosis. The AASLD does not recommend metformin as treatment of MASLD or MASH, although it is an important therapeutic option in patients with coexisting T2DM and metabolic syndrome [100,101]. Metformin is still recommended as a first-line agent for patients with T2DM and cardiovascular risk, which has some advantages measurable in reducing hepatic steatosis.

### 4.6. GLP-1 Receptor Agonists

Glucagon-like peptide-1 (GLP-1) receptor agonists are a promising new therapeutic potential of metabolic diseases, including MASLD, type 2 diabetes mellitus (T2DM) and cardiovascular disorders [104]. In addition to improving glycemic control, these agents yield robust benefits on liver health and are now a mainstay in the treatment of individuals with both metabolic diseases [105]. GLP-1 receptor agonists like liraglutide, semaglutide and dulaglutide mimic the actions of a naturally occurring incretin hormone called GLP-1 [106,107]. They facilitate glucose-sensitive insulin release, inhibit glucagon secretion, delay gastric emptying and promote satiety [108,109]. The improvement in insulin sensitivity is one of the most relevant pathways to explain these results. Reduced hepatic glucose production and circulating free fatty acids may reflect improved insulin sensitivity, which is critical for resolving liver fat accumulation [110,111]. GLP-1 receptor agonists (GLP-1 RAs) have been shown to lower MASH disease activity, evidenced by histological improvements in inflammation and hepatocellular ballooning, as well as decreases in liver fat and MASH biomarkers [111]. Furthermore, GLP-1 receptor agonists have demonstrated anti-inflammatory properties that are essential in the pathophysiology of NASH. Because these agents have been shown to exert anti-inflammatory effects by down-regulating pro-inflammatory cytokines [112], they may be expected not just to slow disease progression but also even reverse the damage seen in liver diseases [113]. While an effective treatment targeting individuals with coexistence of MASLD and T2DM would ideally comprise a multifaceted mechanism, the fact that it is able to reduce body weight [114] represents one key factor as obesity in this highly prevalent group forms part of almost every therapeutic issue [110]. In addition to these direct effects, GLP-1 receptor agonists have many actions that would be most beneficial in the setting of MASLD related disease. In recent years, there has been more active research concerning the function of GLP-1 receptor agonists in MASLD therapy and prevention. Available evidence supports the ability of GLP-1 receptor agonists to correct hepatic steatosis and its related inflammatory events. According to a study, which examined the impact of semaglutide in patients with MASLD [115], semaglutide surprised with its results and managed to significantly reduce liver fat content as well as enhance some of the liver enzymes in this respect, but without significant benefits on fibrosis stage improvement [106,116]. In addition, another study shows that treatment with semaglutide resulted in a reduction in the Controlled Attenuation Parameter (CAP) score, E fibrosis score, NAFLD fibrosis score, and fibrosis-4 (FIB-4) score [117,118]. The effects of dulaglutide on metabolic dysfunction-associated steatotic liver disease (MASLD) are not yet well understood, as research is limited. In a post hoc analysis of the AWARD trials (Assessment of Weekly Administration of LY2189265 [dulaglutide] in Diabetes), dulaglutide (1.5 mg once weekly) was found to improve liver enzyme levels compared to placebo, suggesting a reduction in liver fat [119]. Additionally, a Japanese retrospective study showed that dulaglutide (0.75 mg once weekly) significantly improved liver enzyme levels and liver stiffness, as assessed by transient elastography, after 12 weeks of treatment in type 2 diabetes patients with biopsy-confirmed MASH [110]. Liraglutide, currently indicated for obesity or overweight individuals with comorbid conditions like type 2 diabetes, hypertension, dyslipidemia has shown positive effects on liver histology. While weight loss is considered the primary factor contributing to these histological improvements, potential direct effects within the liver itself cannot be ruled out [106,120]. The LEAN trial focused also on liraglutide therapy for MASH [110,120]. Compared to placebo, treatment with liraglutide not only caused weight loss but also improved histological outcomes including lower liver inflammation and fibrosis scores [120]. These results support the addition of GLP-1 receptor agonists in treating patients with MASLD and T2DM. A meta-analysis of six randomized controlled trials (RCTs) from the “Liraglutide Effect and Action in Diabetes (LEAD)” program found that 26 weeks of treatment with liraglutide (1.8 mg/day) led to improvements in alanine aminotransferase (ALT) levels and hepatic steatosis in 4442 patients with type 2 diabetes (T2DM) [121]. The impact of exenatide on liver biomarkers was studied in 217 patients with type 2 diabetes (T2DM). Participants from three placebo-controlled trials entered an open-label extension study, receiving exenatide at 5 to 10 mcg twice daily in combination with metformin and/or sulfonylureas for at least three years. Among those with elevated baseline ALT levels, improvements in both ALT and AST were observed by week 156, with 41% reaching normalized ALT after three years. Of these patients, the 25% who lost the most weight experienced the largest ALT and AST reductions, while the remaining 75% had similar ALT improvements, regardless of weight loss or HbA1C changes [103,122]. In recent years the effect of GLP-1 receptor agonists on gut microbiota has also become an area of interest. Increasing evidence suggests that these agents can also help to modulate gut microbiome composition and thereby improve metabolic health, as well as ameliorating liver inflammation. The gut–liver axis is critical for the pathogenesis of MASLD and therapeutic targeting of this in combination with microbiota modulation using GLP-1R agonists may represent a new avenue for treatment. Given the cardiovascular advantages of GLP-1 receptor agonists adding to their indication for MASLD, T2DM and cardiometabolic derangements [107]. GLP-1 receptor agonists (GLP-1 RAs) have demonstrated effects on endothelial function, including enhanced vasodilation, as well as on intima-media thickness, which contributes to benefits in atherosclerosis management and plaque stability [123]. The American Association of Clinical Endocrinologists’ 2022 Clinical Practice Guidelines for MASLD recommend GLP-1 receptor agonists (GLP-1 RAs) for individuals with type 2 diabetes (T2D) and biopsy-confirmed metabolic-associated steatohepatitis (MASH) or those at high risk of MASH to provide cardiovascular benefits [124,125]. Similarly, the 2023 guidance from the American Association for the Study of Liver Diseases advises using semaglutide in patients with MASH and T2D or obesity, citing its cardiovascular benefits and improvements in related comorbidities [126]. Semaglutide, as was investigated in the SUSTAIN trials, significantly decreased CV events in patients with T2DM. However, the mechanistic pathways for which GLP-1 receptor agonists impart cardiovascular benefit are only emerging [120]. Yet, these have benefits on weight loss and blood pressure lowering and also significantly reduce lipid profiles which in turn are associated with better outcomes of cardiovascular risk [110]. These cardio-protective effects could be quite important in patients with MASLD, who have higher cardiovascular morbidity and mortality. Cross Current Guidelines from the ADA and EASD have also indicated that personalized strategies for people with T2DM should include using GLP-1 receptor agonists as cornerstone therapy in efforts to reduce glycemia driven risk, as well as cardiovascular risks [127]. GLP-1 receptor agonists (GLP-1RAs) with demonstrated cardiovascular benefits may also be suitable for patients with type 2 diabetes (T2D) who do not have established cardiovascular disease (CVD) but exhibit high-risk indicators. This group includes patients aged 55 years or older who have more than 50% stenosis in coronary, carotid, or lower extremity arteries, left ventricular hypertrophy, or a reduced estimated glomerular filtration rate (eGFR) [128].

### 4.7. SGLT2 Inhibitors

Sodium–glucose co-transporter-2 inhibitors (SGLT2 inhibitors) have emerged as important therapeutic agents in the treatment of various metabolic diseases, including metabolic dysfunction-associated steatotic liver disease (MASLD), type 2 diabetes mellitus (T2DM), and cardiovascular-related disorders [129]. These agents work by inhibiting the reabsorption of glucose in the proximal renal tubules to facilitate urinary glucose excretion. This good glycemic control, combined with extra beneficial effects on body weight and upon the cardiovascular system, led to an indispensable significance for these agents as part of our treatment strategies among patients facing this deadly triumvirate of interconnected conditions [130]. The mechanisms of action on MASLD are multiple and complex for the pathophysiological effects of SGLT2 inhibitors. One key way is that they work by enhancing insulin sensitivity. As MAFLD is linked with insulin resistance [131,132], their ability to enhance insulin sensitivity also means that SGLT2 inhibitors may decrease hepatic glucose production and reduce circulating free fatty acid levels [51]. This reduction is essential because an overabundance of free fatty acids leads to substantial hepatic fat overflow and inflammation [133]. SGLT2 inhibitor usage can also have significant extra-hepatic anti-inflammatory effects on the liver. It greatly reduces inflammation through clinical studies of pro-inflammatory cytokine and systemic inflammatory markers too [133]. The observed weight loss following SGLT2 inhibitor treatment is especially useful in the context of T2DM because obesity is a major aggravating factor for both MAFLD and cardiovascular diseases. New research has shed light on the potential role of SGLT2 inhibitors for NAFLD, which have recently been shown to be useful constituents. A meta-analysis and systematic review by Amjad et al. assessed the impact of various SGLT2 inhibitors, such as ipragliflozin, empagliflozin, dapagliflozin, canagliflozin, and luseogliflozin [134]. The results revealed that these SGLT2 inhibitors significantly improve liver health by lowering the levels of aspartate aminotransferase (AST) and alanine aminotransferase (ALT) [134]. A variety of small-scale studies have indicated that dapagliflozin treatment can improve liver enzyme levels, reduce liver fat, and decrease liver stiffness in patients with MASLD [135,136]. In a nonrandomized, open-label study involving 11 patients with biopsy-confirmed metabolic dysfunction-associated steatohepatitis (MASH), a 24-week course of dapagliflozin at 5 mg per day resulted in decreased liver enzyme levels and better metabolic parameters [137]. The EFFECT-II study, which investigated the effects of omega-3 carboxylic acids combined with dapagliflozin on liver fat in diabetic patients, found that a daily dose of 10 mg of dapagliflozin together with omega-3 carboxylic acids significantly reduced liver fat content, as measured by MRI-proton density fat fraction (MRI-PDFF) [110]. Additionally, dapagliflozin alone decreased all markers of hepatocyte injury in individuals with type 2 diabetes (T2DM) and MASLD. In a randomized, active-controlled, open-label trial involving 57 patients with T2DM and MASLD diagnosed by ultrasound, treatment with dapagliflozin at a dose of 5 mg per day for 24 weeks led to improved liver enzyme levels, reduced steatosis, and decreased fibrosis, as evaluated by transient elastography (FibroScan—Echosens, Paris, France) [110,138]. A meta-analysis that reviewed 11 trials with a total of 6745 patients suffering from type 2 diabetes mellitus (T2DM) found that canagliflozin significantly reduced liver enzyme levels. In a randomized controlled trial (RCT), 37 T2DM patients with metabolic dysfunction-associated steatotic liver disease (MASLD) were treated with canagliflozin at a daily dose of 300 mg for 24 weeks, which resulted in lower liver enzyme levels and decreased intra-hepatic triglyceride content, as evaluated using proton magnetic resonance spectroscopy (MRS) [130]. In a similar vein, a small prospective, nonrandomized, open-label study involving a single group showed that administering canagliflozin at a dose of 100 mg per day for 12 months significantly reduced hepatic fat fraction in T2DM patients with MASLD, as measured by MRI [139,140]. Furthermore, in a cohort of nine patients with T2DM and biopsy-confirmed metabolic dysfunction-associated steatohepatitis (MASH), treatment with canagliflozin at 100 mg per day for 24 weeks resulted in notable improvements in the histological characteristics of NASH, including decreases in steatosis, lobular inflammation, ballooning, and fibrosis stage [110]. The ELIFT trial, a prospective, open-label, randomized clinical study initiated by investigators, involved 50 patients with type 2 diabetes mellitus (T2DM) and metabolic dysfunction-associated fatty liver disease (MASLD). The trial demonstrated that treatment with empagliflozin at a dose of 10 mg per day for 20 weeks resulted in significant reductions in liver enzyme levels and liver fat content, as assessed by MRI-proton density fat fraction (MRI-PDFF) [110]. A 2020 study by Kahl et al. involved 84 patients with diabetes who had excellent glycemic control, randomly assigned to either empagliflozin or a placebo [141]. MRI assessments revealed that patients on empagliflozin experienced improved liver fat content, indicating that the benefits of SGLT-2 inhibitors on hepatic steatosis may involve mechanisms beyond glycemic control and weight loss alone [141]. In addition to improving liver health, SGLT2 inhibitors are also known for their robust cardiovascular benefits. Results from the landmark EMPA-REG OUTCOME trial demonstrated a reduced risk of major adverse cardiovascular events in patients with T2DM and established CVD receiving empagliflozin [142,143]. This evidence has driven the inclusion of SGLT2 inhibitors in clinical guidelines as a key component for patients with both diabetes and CV risk predicates. They also display a loop-like diuretic effect that contributes to lower blood pressure and body weight, along with an increase in hematocrit through mechanisms that remain unclear. These metabolic and hemodynamic impacts of SGLT2 inhibitors have shown considerable advantages in preventing cardio-renal events, as highlighted by the results of the EMPA-REG OUTCOME trial [110]. Due to their cardiovascular benefits, SGLT2 inhibitors are now recommended in the guidelines of key international diabetes associations (including the ADA and EASD) as second-line therapy for patients with T2DM who have established atherosclerotic CVD or high risk for cardiovascular events. Recommendations from EASD guidelines highlight SGLT2 inhibitors for the treatment of MAFLD as one way to positively impact liver outcomes concerning liver enzymes and steatosis [2]. SGLT2 inhibitors are used to manage type 2 diabetes, chronic kidney disease, and heart failure by obstructing SGLT2 in the proximal tubule. These drugs have a modest diuretic effect, and the increase in hemoglobin was first attributed to hemoconcentration. However, increased reticulocyte count and hepcidin suppression pointed to a different pathophysiology. It is well-known that diabetic kidneys are prone to tubulointerstitial hypoxia. This implies that SGLT2 inhibitors could enhance hypoxia by alleviating the workload on the proximal tubule, resulting in an increase in hemoglobin levels [144]. However, the use of SGLT-2 inhibitors should be approached with caution due to potential side effects, including diabetic ketoacidosis and urogenital infections. It is strongly advised to regularly monitor liver and kidney function [145].

### 4.8. Combination Therapy GLP-1 Receptor Agonists/SGLT2 Inhibitors

A new pharmacotherapy approach in managing type 2 diabetes mellitus (T2DM) suggests using a combination of GLP-1 receptor agonists and SGLT2 inhibitors for patients who have T2DM along with nonalcoholic fatty liver disease (NAFLD) and a considerable cardiovascular risk [146,147]. SGLT2 inhibitors and GLP-1 receptor agonists are expected to play a key role in treating patients with type 2 diabetes who also have NASH [148]. The two drug classes offer complementary benefits with GLP-1 RAs focusing more on glycemic effects and weight loss, whereas SGLT2-inhibitors offering combined glucose lowering as well as cardiovascular morbidity reduction [149]. Through the combination of these therapies, patients who have complex metabolic conditions could potentially benefit from a broad spectrum treatment approach that not only manages hyperglycemia but also excess weight and fat in liver leading to cardiovascular risks. More specifically, the combination therapy not only improves the control of blood glucose, blood pressure, and body weight, but also might act to minimize the evolution and progression of diabetic nephropathy [149]. DURATION-8 was one of the earlier trials to evaluate a GLP1 RA (exenatide) with an SGLT2 inhibitor (dapagliflozin). In this trial, patients with T2DM inadequately controlled on metformin were randomized to receive exenatide alone; or dapagliflozin monotherapy; or the two in combination. Results demonstrated greater HbA1c reductions for patients receiving the combination therapy than those who received exenatide alone or dapagliflozin alone, with a mean reduction of 2.0% vs. 1.6%, and 1.4%, respectively In addition, a combination approach was behind the biggest weight loss, which more than doubled that seen with either agent alone versus 1.54% and 2.22% for exenatide or dapagliflozin monotherapy, respectively [150]. The potential of these two agents combined together is further supported by the AWARD-10 trial (which evaluated dulaglutide in combination with either empagliflozin or dapagliflozin, as SGLT2 inhibitors). The combination was associated with a greater decrease in HbA1c compared to SGLT2 inhibitors alone, as the mean reduction of 1.34% observed for patients on the fixed-ratio regimen versus 0.82% seen among those treated with monotherapy clearly demonstrated [147]. More importantly, the combination also achieved a more pronounced reduction in weight with patients losing an average 2.5 kg rather than minimal changes seen for SGLT2 inhibitors alone. As weight loss is a major contributor to decreasing liver fat and ameliorating outcomes in MASLD, the data emerging from AWARD-10 study are yet another additional form evidence reinforcing benefits of GLP1 RAs combined with SGLT2 inhibitors for those individuals having T2DM accompanied by MASLD [147]. The SUSTAIN-9 trial evaluated semaglutide (GLP-1 RA) in patients who were already on treatment with an SGLT2 inhibitor. Adding semaglutide was associated with significantly better glycemic control: mean HbA1c decreased 1.5%, vs. only a 0.8% decrease in the placebo arm, which fell to non-significance (*p* < 0.0001). In addition, patients on combination therapy lost significantly more weight (4.7 kg) than those on placebo-group (1.2 kg). While the trial was not targeting MASLD specifically, its reduction in body weight and HbA1c indicate likely positive effects on liver fat decrease and composite endpoints for metabolic dysregulation in this condition [151,152]. The latest care guidelines for type 2 diabetes mellitus (T2DM) patients from the American Diabetes Association (ADA) and the European Association for the Study of Diabetes (EASD) are now focusing more on tailoring treatment strategies to each individual, especially for patients with coexisting cardiovascular or liver-related conditions [153].

### 4.9. Pioglitazone

Pioglitazone has become an established and crucial part of treatment for metabolic dysfunction-associated steatotic liver disease (MASLD) but also in the management of coronary artery diseases along with diabetes [154]. The main mode of action of pioglitazone is through activation of the peroxisome proliferator-activated receptor gamma (PPAR-γ), a master regulator for glucose and lipid homeostasis [155]. As an agonist at PPAR-γ, pioglitazone increases insulin sensitivity. Further, it helps in normalizing lipid metabolism by lowering the levels of circulating free fatty acids that is essential for correction hepatic steatosis. It can also modulate inflammation and adipocyte differentiation, which may normalize the metabolic characteristics of adipocytes. Clinical studies have recently verified the strength of pioglitazone to treat MASLD. Treatment with pioglitazone for one year, even at a low dosage, significantly improved liver steatosis and inflammation while also enhancing systemic and adipose tissue insulin resistance in patients with type 2 diabetes (T2D). Notably, the beneficial effects of pioglitazone on metabolic dysfunction-associated steatotic liver disease (MASLD) were found to be independent of blood glucose control [156]. The PIVENS study of pioglitazone in MASH and T2DM is a landmark trial. This randomized, controlled trial determined that pioglitazone improved liver histology to a significantly higher degree than placebo [157]. MASH patients showed an incidence of a histological improvement with protection occur from 43% in these who received pioglitazone [157]. Another key trial is the FLINT study that investigated pioglitazone in non-diabetic MASH patients. Pioglitazone treatment resulted in significant improvements in liver histology including steatosis, inflammation and ballooning degeneration. A consistent improvement was also noted when the anti-inflammatory effects of pioglitazone were taken into account at molecular level.This observation suggests the effectiveness of pioglitazone not only in diabetics patients but also in non-diabetic MASLD cohort, which potentially expand its clinical application or utility. Improvement in insulin sensitivity is the most important attribute of pioglitazone, particularly for patients with MASLD [158]. The reduction in CVD risk with pioglitazone has been demonstrated by multiple trials of T2DM. And the PRO-active study, a landmark trial demonstrated that pioglitazone reduced MACE (defined as myocardial infarction and stroke) in patients with type 2 diabetes and established cardiovascular disease [159]. This protection of the cardiovascular system is due to a number of mechanisms, including improved lipid profiles, attenuation in blood pressure and improving endothelial function. The lipid-modulating effects of pioglitazone were demonstrated in the Pioglitazone Effect on Regression of Intravascular Sonographic Coronary Obstruction Prospective Evaluation (PERISCOPE) and the Carotid Intima-Media Thickness in Atherosclerosis Using Pioglitazone (CHICAGO) trials [160]. In the PERISCOPE trial, there was a significant increase in high-density lipoprotein cholesterol (HDL-C) levels, along with a decrease in triglyceride (TG) levels among 543 patients with type 2 diabetes mellitus (T2DM) [161]. The cardiovascular risk profile of pioglitazone continues to be a topic of discussion, especially regarding the risk of heart failure associated with weight gain and fluid retention. Pioglitazone appears to have a positive impact on alleviating metabolic dysfunction-associated steatotic liver disease (MASLD), as indicated by consistent improvements in histopathology, liver enzyme levels, HOMA-IR, and reductions in blood lipid levels in both non-diabetic and diabetic MAFLD patients. The treatment was mostly well tolerated, although a higher occurrence of edema was noted in the pioglitazone group among diabetic MASLD patients [162].

### 4.10. DPP-4 Inhibitors

Incretin hormone-targeting dipeptidyl peptidase-4 (DPP-4) inhibitors are an important class of agents indicated for the management of type 2 diabetes mellitus T2DM that owe their significant glucose-lowering effect to up-regulated endogenous incretin levels [110]. DPP-4 inhibitors block the enzyme that breaks down incretin hormones leading to increased insulin secretion, decreased glucagon release and lower blood glucose. In addition, DPP-4 inhibitors may ameliorate dyslipidemia and inflammation as well as weight reduction [163,164]. DPP-4 lowers the levels of active glucagon-like peptide 1 (GLP-1), which is associated with the onset of MASLD. These issues support the hypothesis that DPP-4 inhibitors may improve the histological aspects of steatohepatitis and fibrosis, potentially helping to halt disease progression [163,164]. Some of the most commonly prescribed DPP-4 inhibitors include sitagliptin, linagliptin, vildagliptin, saxagliptin, and alogliptin, all of which are widely available globally [165]. As the first DPP-4 inhibitor developed, sitagliptin has been extensively studied for its effectiveness in treating NAFLD/NASH. However, findings regarding its impact on MASLD have been variable. An observational pilot study involving 15 patients with type 2 diabetes mellitus (T2DM) and biopsy-confirmed MASH demonstrated that a one-year treatment with sitagliptin (100 mg/day) led to improvements in liver enzymes, hepatocyte ballooning, and MASH scores [166,167]. Additionally, a prospective, 24-week, single-center, open-label comparative study with 20 Japanese patients suffering from T2DM and MAFLD showed a significant reduction in intrahepatic lipid content after 34 weeks of sitagliptin treatment [168]. In contrast, a randomized controlled trial (RCT) involving 50 patients with MASLD indicated that there were no improvements in liver enzymes or liver stiffness, measured by magnetic resonance elastography, after 24 weeks of sitagliptin (100 mg/day) [169] whereas it was observed that gemigliptin reduced the succinate-induced production of mitochondrial and intracellular reactive oxygen species (ROS) [169]. Furthermore, a separate study involving 44 patients with T2DM and biopsy-confirmed MASLD found that sitagliptin treatment (50 mg/day) over 12 months did not reduce liver enzymes, although it did achieve a 0.7% decrease in HbA1c [170]. Multiple trials have indicated that vildagliptin may positively impact MASLD. In one study with 44 patients diagnosed with type 2 diabetes mellitus (T2DM) and hepatic steatosis, treatment with vildagliptin (50 mg twice daily) for six months led to decreased liver enzymes and reduced intrahepatic triglyceride levels, as assessed by MRI [171]. In a randomized controlled trial carried out in Pakistan, administering vildagliptin (50 mg twice daily) for a duration of 12 weeks led to improvements in liver enzymes and a decrease in steatosis grading, as assessed by ultrasound [172]. The SAVOR-TIMI 53 trial is the first cardiovascular outcome study of saxagliptin to show no increased risk over placebo for major adverse cardiac events. This is clinically important as the majority of patients with T2DM have concomitant cardiovascular disease and require treatment that they can take long term [173]. In college students of the TECOS trial, with regard to cardiovascular protection, no change was recognized from a imminent-end result standpoint between sitagliptin-treated patients and also people receiving the placebo [174]. Contemporary clinical guidelines also highlight a potential utility of DPP-4 inhibitors in the treatment canonical T2DM, especially with coexisting MASLD and cardiovascular disease. Although there is growing in vivo and in vitro evidence suggesting the potential benefits of DPP-4 inhibitors for NAFLD treatment, there are only a few clinical trials demonstrating significant efficacy in affected patients. Consequently, DPP-4 inhibitors are not yet widely endorsed for the treatment of MASLD [175].

### 4.11. Bariatric Surgery

Bariatric surgery is considered a highly effective option for weight loss and managing Type 2 Diabetes (T2D) in individuals with severe obesity [176]. Furthermore, metabolic surgery helps decrease ectopic fat, especially in the liver, resulting in notable improvements in the symptoms of metabolic dysfunction-associated steatotic liver disease (MASLD) and metabolic dysfunction-associated steatohepatitis (MASH) [177,178]. Studies have shown that patients achieve significant reductions in steatosis after undergoing bariatric surgery, with 80% of individuals resolving MASH within the first year following the procedure. This improvement correlates with better insulin resistance [176,179]. Although there are observed improvements in MASH histology following bariatric surgery, the 2018 AASLD guidelines indicate that it is too early to recognize foregut bariatric surgery as a validated treatment option for MASH [180]. The effectiveness of bariatric surgery in treating MASLD is still under discussion because there are no randomized trials available. However, bariatric surgery has the potential to achieve sustained weight loss of up to 30% and may also lead to the resolution of MASLD [126,181]. The main types of bariatric surgery currently include Roux-en-Y gastric bypass, sleeve gastrectomy, biliopancreatic diversion with duodenal switch, and device implantation. More than half of patients who undergo bariatric surgery experience the resolution of MASH, and 30% to 40% demonstrate improvements in liver fibrosis [182]. In a study involving individuals with obesity and MASLD, bariatric endoscopy was found to enhance both analytical and ultrasound measurements related to insulin resistance, hepatic fat levels, and hypertriglyceridemia [183]. This intervention has demonstrated effectiveness in reducing mortality, cardiometabolic risks, and liver disease [184]. Nevertheless, the exact mechanisms driving the swift metabolic improvements, especially in glucose and lipid balance, remain unclear. Bariatric surgery lowers type 2 diabetes mellitus (T2DM) rates by targeting multiple facets of the disease. It enhances glucose control and triggers diabetes remission in 95–100% of patients. Originally, these benefits were thought to stem mainly from weight loss [184]. Bariatric surgery reduces the risk of major vascular diseases by markedly decreasing blood triglyceride and glucose levels. Following the procedure, levels of postprandial adiponectin, GLP-1, insulin, and serum insulin-like growth factor 1 significantly rise. Elevated adiponectin is linked to lower total fat mass and a decreased risk of atherosclerosis. Specifically, laparoscopic sleeve gastrectomy can result in substantial weight loss and effective blood pressure control in obese patients. Studies show that within the first year after surgery, hypertension remission rates are between 60% and 70%, potentially reaching as high as 90% with extended follow-up [184]. Surgery is generally safe and beneficial for higher-morbidity patients, even those with well-compensated cirrhosis [185].

### 4.12. Other Therapeutic Targets

**Figure 4:**
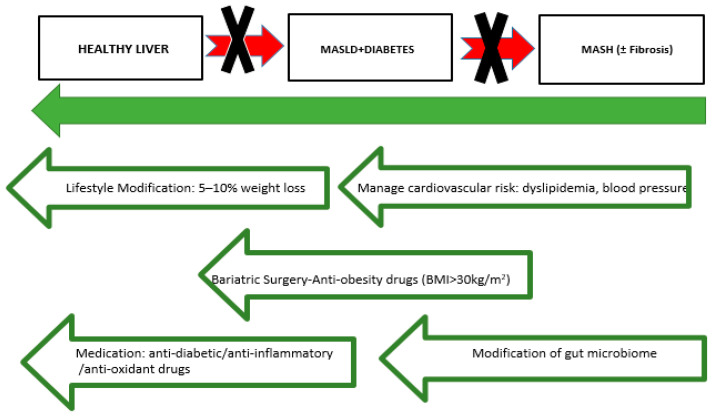
General management of MASLD (MASLD: Metabolic dysfunction-associated steatotic liver disease, MASH: Metabolic Dysfunction-Associated Steatohepatitis and BMI: body mass index).

There are multiple novel drug classes under investigation for the treatment of MASLD and MASH, notably drugs that target metabolic pathways, anti-inflammatory factors or fibrotic–fibrogenic effects. Fibroblast growth factor 19 (FGF19) is released by intestinal cells upon FXR activation and, after traveling to the liver via the portal vein, it regulates bile acid metabolism through the FGF receptor 4/β-klotho complex. FGF19 also plays a role in lipid and glucose metabolism. However, it can drive tumorigenesis through the IL-6/STAT3 pathway. NGM282, a newly engineered analog of FGF19 designed to avoid activation of the STAT3 pathway and thus likely reduce the tumorigenic potential of FGF19, showed a significant reduction in liver fat content in a phase II study with 82 MASH patients [1]. Cenicriviroc acts as a dual antagonist for the C-C chemokine receptors CCR2 and CCR5, both of which are essential for the recruitment and differentiation of macrophages and are associated with the onset of MASH. A substantial two-year phase II clinical trial investigated the safety and effectiveness of cenicriviroc in treating MASH in adults with liver fibrosis, enrolling 289 patients. After one year, the study observed a significant reduction in systemic inflammation; however, this did not translate into a definitive improvement in MASH [186]. Carnitine helps regulate the transport and oxidation of free fatty acids within mitochondria and has antioxidant properties in liver cells. In patients with MASH, a 24-week course of L-carnitine improved liver enzyme levels and liver histology [187]. Similarly, in patients with type 2 diabetes and MASLD, a 12-week regimen of the carnitine-orotate complex (824 mg, three times daily) led to reduced serum ALT levels and decreased liver fat, as evaluated by CT, in a sample of 78 patients (25877813). Elafibranor acts as a dual agonist for PPAR-α and PPAR-δ, supporting improvements in insulin resistance in both liver and peripheral tissues [188]. In a 52-week phase 2b trial, daily administration of elafibranor showed a tendency to resolve MASH without progressing fibrosis, although some methodological limitations were present [189]. Oltipraz is an LXRα inhibitor with antisteatotic properties, reducing liver fat by inhibiting lipogenesis and enhancing lipid oxidation. A phase 2 study (NCT01373554) assessed oltipraz’s efficacy and safety in Asian patients with ultrasound-confirmed MASLD, each with over 20% hepatic fat content [190]. Resmetirom, a recently FDA-approved drug, is currently the only available treatment for non-cirrhotic MASH. It acts as a selective thyroid hormone receptor-β agonist, aimed at improving MASH by enhancing hepatic fat metabolism and lowering lipotoxicity [191,192]. This treatment resulted in a significant reduction in hepatic fat after 12 weeks and 36 weeks of treatment in patients with NASH. Lanifibranor is a pan-PPAR agonist evaluated in the Phase II NATIVE trial, which demonstrated significant results in achieving NASH resolution, reversing fibrosis, and improving both lipid profiles and insulin sensitivity in adult patients with non-cirrhotic MASH after just 24 weeks of treatment [193]. Saroglitazar is a PPARα/γ agonist demonstrated to improve outcomes in MASLD/MASH. Its dual-sensitizing action enhances β-oxidation and reduces triglyceride synthesis while also increasing insulin sensitivity [194]. Recently, herb-derived polysaccharides, derived from plants, mushrooms, seaweeds, have been recognized for their diverse pharmacological activities, which include hypoglycemic, hypolipidemic, antioxidant, anti-inflammatory, and immunomodulatory effects. They also improve insulin resistance and help regulate glucose and lipid metabolism. Consequently, these polysaccharides have attracted considerable attention as natural agents with significant potential for addressing metabolic diseases [195,196]. Additionally, they can benefit MASLD by reducing hepatic lipid accumulation and steatosis, safeguarding mitochondrial function, and alleviating hepatic oxidative stress and inflammation [197]. Vitamin D deficiency is a prevalent global concern, with low serum levels of 25(OH)D3 found in both adults and children diagnosed with NASH. There is a proposal that addressing vitamin D3 deficiency may aid in preventing or treating the progression from MASLD to MASH. However, as of now, no large placebo-controlled randomized trials have been carried out to substantiate this strategy [198]. MSDC-0602K is a novel thiazolidinedione that functions as a mitochondrial pyruvate carrier (MPC) inhibitor, which reduces its direct interaction with PPARγ. MSDC-0602K replicates the effects of first-generation insulin sensitizers by inhibiting MPC in hepatocytes, resulting in lower gluconeogenesis and improved management of hypoglycemia following prolonged fasting, without affecting other hepatic responses to fasting. Research involving diet-induced obese db/db mouse models showed that MSDC-0602K effectively reduced insulin resistance, lipogenesis, and gluconeogenesis, while also promoting lipid oxidation in the liver, similar to the effects of pioglitazone [199]. Long-term antibiotic use can reduce gut microbiota diversity and lower liver inflammation [200]. Rifaximin, an antibiotic that is not absorbed in the intestines, has demonstrated significant reductions in AST, ALT, low-density lipoprotein (LDL), and BMI among patients with NASH [201,202]. Prebiotics, which are partially digested food components, foster beneficial alterations in gut microbiota. The administration of prebiotics serves as an effective therapeutic strategy for MASLD by increasing the abundance of probiotics in the gut. This approach may exert its effects through multiple mechanisms, such as decreasing oxidative stress, reducing inflammation, enhancing glucose tolerance, and lowering triglyceride accumulation by modifying gut microbiota. Moreover, prebiotics promote the production of short-chain fatty acids (SCFAs), which are beneficial for managing MASLD [51]. In the following Figure 4 and Table 2, are given briefly information about the therapeutic option for the prevention of MASLD and cardiovascular events.

## 5. Discussion

NAFLD-MASLD has become a major public health issue around the world. Its widespread prevalence is strongly associated with lifestyle changes and growing rates of metabolic conditions such as obesity and type 2 diabetes [51]. Studies have shown that patients with MASLD are at higher risk of cardiovascular morbidity and this is mainly because of metabolic dysfunction [203]. Liver fat content is associated with risk factors for cardiovascular disease including dyslipidemia elevated blood pressure and increased arterial stiffness. Increasing the cardiovascular (CV) stressors that are already present, T2DM further worsens these CV risks and begins a cascade of metabolic distress [204]. The strong association between MASLD and CVD and T2DM implies that approaches to the therapy management should be viewed as part of strategies for cardiovascular health and diabetes control. As such, an integrated care model is needed to better manage the health outcomes and long-term complications shared by these comorbid conditions. Treatment of the triad is more a matter or modifying lifestyle habits. Incorporating a healthy Mediterranean diet with healthy fats, whole grains and high fruit/vegetable intake has been shown to be effective at reducing liver fat, as well as improve markers of metabolism. Regular physical activity including a combination of aerobic and resistance training is also essential in increasing insulin sensitivity, as well as weight reduction. The enhancement of type 2 diabetes and MASLD (especially fibrosis) is connected to the success of obesity management [205]. In addition to lifestyle changes, management of patients with MASLD also requires pharmacological interventions due to the strong association between MASH and CVD as well as T2DM. For example, the first-line medication for T2DM, metformin has been shown to improve liver enzymes and histology among MASLD patients. Statin drugs have been approved for use in MASLD patients. Moreover, anti-hypertensive treatment should be individualized to the patient considering agents with additional metabolic properties providing a pan-genotypic management plan in multi-morbid patients. However, as recently revealed by a series of clinical trials and observational studies, other newly proposed potential benefits in the secondary prevention arena include cardio-protective actions similar to that seen with lipid-lowering therapy for which these patients are already at high priority. Over the last decade, newer pharmacological agents have emerged as effective treatments which not only improve glycemia but also exhibit cardiovascular benefits while possessing organ-protective properties over liver dysfunction [206]. DPP-4 inhibitors, despite their initial promise, have not consistently improved liver steatosis and fibrosis, resulting in their lack of recommendation for MASLD. In contrast, pioglitazone, SGLT2 inhibitors, and GLP-1 receptor agonists have shown strong effectiveness in reducing liver fat and resolving MASH, positioning them as promising treatments for MASLD [16]. Importantly, pioglitazone is the only drug approved for MASH with significant liver fibrosis by all major liver societies [207,208]. Both SGLT2 inhibitors and GLP-1 receptor agonists have demonstrated notable efficacy, with SGLT2 inhibitors being more effective in the only head-to-head study conducted so far. However, GLP-1 receptor agonists are not yet approved for non-diabetic patients, and SGLT2 inhibitors are restricted to non-diabetic patients with heart or renal failure. This underscores the critical need for further trials to evaluate these medications in MASLD patients without T2DM. SGLT2 inhibitors and GLP-1 receptor agonists are expected to play a significant role in treating type 2 diabetes patients with MASH. While several studies, such as AWARD 10, Duration 8, and AGATE, have looked at the combined use of SGLT2 and GLP-1 receptor agonists in T2D patients, there have not yet been any studies specifically focused on the effectiveness of this combination for MASH treatment [147]. DPP-4 inhibitors, despite their initial promise, have not consistently improved liver steatosis and fibrosis, resulting in their lack of recommendation for MASLD. In contrast, pioglitazone, SGLT2 inhibitors, and GLP-1 receptor agonists have shown strong effectiveness in reducing liver fat and resolving NASH, positioning them as promising treatments for MASLD. Importantly, pioglitazone is the only drug approved for MASH with significant liver fibrosis by all major liver societies. Although a variety of treatment options exist, challenges remain in selecting therapy regimens that effectively treat patients with this triad [209]. Some patients may benefit more from aggressive lifestyle interventions than pharmacological treatment but all in all a drug that not only enhances MASH and/or fibrosis but also aids in weight loss, improves lipid levels, enhances glycemic control, or leads to other cardiometabolic benefits could offer significant added advantages [210], potentially shaping its role in future treatment options. Notably the present therapeutic options available for MAFLD are insufficient since there are no approved drugs for the treatment of this disease. This highlights the importance of developing new therapies focusing on MASLD as well as its associated troubling metabolic conditions in an effective and more beneficial way. At present, the guidelines for the treatment of MASLD mostly value lifestyle changes. Although such approaches have been effective in managing encompassing improvement of metabolic disturbances and hepatic droplet deposition, their results are highly patient dependent and most of the time unavailable in candidates with a more severe illness. These pharmacological agents like are often used to treat each dysfunction within metabolic syndrome, but they do not prevent or improve the states of the liver or cut back on fibrotic development within it. Furthermore, the use of several medications to treat a single disease, which often pertains to the individual comorbidity, creates a situation where polypharmacy is more evident thus bringing about negative outcomes and non-adherence to treatment on the part of the patient. Hence, there is an urgent and great need for managements directed at MASLD and other disorders in patients suffering from metabolic syndrome. The development of MASLD is not a simple or one-dimensional process but rather has many components which include insulin resistance, excess lipid deposition in the liver, inflammation, and development of scar tissue fibrosis. It is preferable to use combinations of drugs that aim at different pathogenic mechanisms for greater liver function, improvement of glucose regulation, reduction in body weight, and cardiovascular status. Emerging evidence indicates that certain pharmacological classes, GLP-1 receptor agonists, dual PPAR agonists and FXR agonists, for example, are promising solutions in the treatment of these different targets. However, while the initial information is optimistic, large controlled trials are necessary to confirm the long-term effectiveness and safety of these compounds in treating MASLD patients with metabolic syndrome. It should be borne in mind that future clinical trials will be broader than naive ‘treat the patient’ ones and will be based on the network of pathophysiological processes including the consequences of the disease such as systemic effects in illnesses like MASLD. Beyond the standard liver-targeted outcomes such as steatosis, transaminase and fibrosis rates, trials should also include assessment of improvements in insulin resistance, dyslipidemia, blood pressure management and cardiovascular risk. Last but not least, the limiting of side effects and events will be very important to this kind of patients, especially because often people suffering from metabolic syndrome have several other conditions that can worsen the side effects of the treatment. Through a thorough analysis of the risks and benefits of new treatments, subsequent studies will make certain that all the positive effects of treatment will cover various metabolic parameters with little detrimental effects on the patients. In addition, effective treatment is hampered by the large variability of MASLD in its manifestations in individual patients with metabolic syndrome. NAFLD ranges from simple steatosis and metabolic dysfunction-associated steatohepatitis to cirrhosis and each stage has a different prognosis and treatment requirements. It is necessary for future studies to focus more on classifying patients in terms of the disease stage, risk factors, and metabolic subtypes to address the question of which subgroups are more responsive to the intervention espoused. Kinetic advancements, for instance the introduction of biomarker-driven patient stratification and imaging approaches, should enhance the precision patient selection for the treatment outcomes, thus making it possible to treat MASLD in a precision medicine way. The advent of such multi-target therapies could most likely deleverage the burden of polypharmacy which is generally faced in this population. Considering both the liver specific and systemically acting features of metabolic syndrome in one treatment would favor more compliance within the patient population and help alleviate the problem of many medications outlasting a long treatment period which is crucial for effective management. Lastly, it is important to mention that efforts conducted in parallel hepatology, endocrinology, cardiology, and pharmacology will help to better understand the processes that lead to, and new mechanisms of MASLD. Therefore, the focus needs to be put on conducting integrated clinical trials developed in order to analyze the specific pharmacotherapy approaches that are safe, effective, and multifaceted. Cutting across these converging lines is the potential to improve liver health, control its associated metabolic disorders and even reduce cardiovascular risk to abolish or achieve the management of MASLD in a paradigm shift. Whether these are possible will depend on the extent of integration of new drugs into existing treatments and pharmacological management.

## 6. Conclusions

The management of patients with MASLD, CVD, and T2DM requires a multifaceted and holistic strategy that encompasses lifestyle interventions, pharmacotherapy, and diligent monitoring of cardiovascular risk factors. By addressing the interconnected nature of these conditions, healthcare providers can significantly enhance patient outcomes, reduce the risk of complications, and improve the quality of life for individuals grappling with these chronic diseases. In the ever-evolving world of these intricately interwoven healthcare processes, it remains paramount that we sustain our vigilance toward developing optimal integrated care pathways to address and manage the multifaceted nature associated with our patient population. Such studies are warranted, as these diseases share a considerable amount of similarities in their underlying structural changes and common inflammation pathways. Overall, as the challenges of MASLD, CVD and T2DM are huge and demanding, a multi-disciplinary and cross-sectoral sensible strategy is needed in order to devise arrangements that would involve changes for better long-term health among patients.

## Figures and Tables

**Table 1:** Lipid-lowering therapies in liver steatosis.

	Steatosis	Liver Enzymes	Inflammation	CV Risk
PUFAs	Decreased	Decreased	Decreased	Decreased
Statins	Decreased	Unknown/Increased	Decreased	Decreased
Fibrates	Unknown/Unchanged	Unknown/Unchanged	Decreased	Decreased

CV: cardiovascular; PUFAs: polyunsaturated fatty acids.

**Table 2:** Available therapeutic choices for liver steatosis.

		Weight	Steatosis	Liver Enzymes	CV Risk	Inflammation	Side Effects
Metformin	TONIC (173 patients) (Randomized double-blind, double-dummy, placebo-controlled trial) (1000 mg metformin/day, 800 IU Vitamin E/Day for 96 weeks) HOME (745 patients) (Randomized, placebo-controlled trial) (850 mg metformin/day, for 16 weeks)	Decreased	Decreased	Decreased /Unchanged	Decreased	Decreased	Gastrointestinal effects, lactic acidosis
GLP-1 RAs	AWARD (810 patients) (Open-label, randomized trial) (Dulaglutide 1.5 mg/week, dulaglutide 0.5 mg/week, insulin glarine daily for 52 weeks) LEAN (52 patients) (Double-blinded, placebo-controlled randomized trial) (Liraglutide 1.8 mg/daily for 48 weeks) SUSTAIN9 (302 patients) (Randomized, placebo-controlled trial) (Escalating to 1 mg semaglutide for 30 weeks)	Decreased	Decreased	Decreased	Decreased	Decreased	Gastrointestinal effects, pancreatitis, risks for cancer
SGLT2 inhibitors	EFFECT II (84 patients) (Double-blind randomized placebo-controlled) (Dapagliflozin 10 mg/day + 4 g omega-3 carboxylic acids for 12 weeks) E-LIFT (50 patients) (Randomized controlled trial) (Empagliflozin 10 mg/day > 12 weeks) EMPA-REG-OUTCOME (7020 patients) (10–25 mg empagliflozin/daily for 2.6 years)	Decreased	Decreased	Decreased	Decreased	Decreased	Glycosuria, cardiovascular concern, ketoacidosis, hypotension, bone fracture
DPP-4inhibitors	SAVOR-TIMI (16,492 patients) (Randomized, double-blind, placebo-controlled trial) (Saxagliptin 2.5–5 mg/day for 18 months)	Unchanged	Decrease/Unchanged	Decreased/ Unchanged	Decreased /Unchanged	Unchanged	Pancreatitis, risks for cancer, acute hepatitis and kidney impairment
Thiazolidinediones	Pro/active (952 patients) (Randomized study) (15–45 mg/day pioglitazone depending on tolerance) PERISCOPE (543 patients) (Randomized controlled trial) (15–45 mg pioglitazone/day for 18 months)	Increased	Decreased	Decreased/ Unchanged	Decreased	Decreased	Hepatitis, bladder cancer, water retention and weight gain
Bariatric Surgery		Decreased	Decreased	Decreased/ Unchanged	Decreased	Decreased /Unchanged	Depending on the type of surgery: indigestion, sagging skin, stomach ulcers, nausea, gall stones

CV: cardiovascular, GLP-1 RAs: glucagon-like peptide-1 receptor agonists, SGLT2 inhibitors: sodium–glucose cotransporter-2 inhibitors, and DPP-4inhibitors: dipeptidyl peptidase 4 inhibitors.
